# Behavioral observations, heart rate and cortisol monitoring in horses following multiple oral administrations of a cannabidiol containing paste (part 2/2)

**DOI:** 10.3389/fvets.2023.1305873

**Published:** 2024-01-03

**Authors:** Fabienne Eichler, Anna Ehrle, Marc Machnik, Katharina Charlotte Jensen, Sabrina Wagner, Natalie Baudisch, Julia Bolk, Magdalena Pötzsch, Mario Thevis, Wolfgang Bäumer, Christoph Lischer, Mechthild Wiegard

**Affiliations:** ^1^Equine Clinic, Veterinary Hospital Freie Universität Berlin, School of Veterinary Medicine, Freie Universität Berlin, Berlin, Germany; ^2^Center for Preventive Doping Research, Institute of Biochemistry, German Sport University Cologne, Cologne, Germany; ^3^Institute of Veterinary Epidemiology and Biostatistics, School of Veterinary Medicine, Freie Universität Berlin, Berlin, Germany; ^4^Institute of Pharmacology and Toxicology, School of Veterinary Medicine, Freie Universität Berlin, Berlin, Germany; ^5^Institute of Animal Welfare, Animal Behavior and Laboratory Animal Science, School of Veterinary Medicine, Freie Universität Berlin, Berlin, Germany

**Keywords:** behavior, CBD, equine, FaceSed, heart rate variability, Horse Grimace Scale, novel object test, sedation score

## Abstract

As a remedy against stress and anxiety, cannabidiol (CBD) products are of increasing interest in veterinary medicine. Limited data is available describing the actual effectiveness of CBD in horses. The aim of this study (part 2 of 2) was to analyze stress parameters via behavioral observation, heart rate monitoring and assessment of blood and saliva cortisol levels in healthy horses treated repeatedly with a CBD containing paste. Twelve horses were randomly assigned to a treatment or a control group. Two pastes were orally administered in a double-blinded study design, one paste containing CBD and one paste without active ingredient. Both pastes were administered twice daily over 15 days (dose: 3 mg CBD/kg). Behavioral observations were conducted daily using a sedation score and a rating of facial expressions, based on the previously described facial sedation scale for horses (FaceSed) and the Horse Grimace Scale. Blood and saliva samples were obtained regularly to determine cortisol levels throughout the study. Cortisol levels were analyzed by means of liquid chromatography/tandem mass spectrometry (LC/MS/MS). Behavioral observations and cortisol levels were compared between groups. Prior to paste administration, a novel object test was performed and the horses’ reaction to loading on a trailer was recorded. Both tests were repeated after 13 days of paste application. Movement patterns such as different gaits during the novel object test were evaluated and an ethogram was designed to assess exhibited behavioral traits. Cardiac beat-to-beat (R-R) intervals were recorded throughout and evaluated using heart rate (HR) and heart rate variability (HRV) parameters. Blood and saliva samples for cortisol analysis were taken before and after the tests. Daily behavioral observations and cortisol levels did not differ between the treatment and the control group. Similarly, analysis of movement patterns, HR, HRV and cortisol levels during the novel object test and trailer test did not identify significant differences between the groups. Regularly administered oral CBD (3 mg/kg BID over 15 days) had no statistically significant effect on behavioral observations, cortisol levels, HR and HRV in horses. Further research is required to establish adequate doses and indications for the use of CBD in horses.

## 1 Introduction

Supplements containing cannabis compounds have been promoted as remedies for the treatment of numerous conditions such as anxiety or osteoarthritis in human and animal patients (1–5). Their popularity has increased in recent years but few scientific studies have investigated the actual effectiveness in animals and specifically horses (6–8). The predominant cannabis compounds include the phytocannabinoids cannabidiol (CBD) and Δ^9^-tetrahydrocannabinol (THC), which is known for its psychoactive properties (9–11). CBD is currently under investigation for its proposed relaxing and anxiolytic effects in humans, rodents and dogs (3, 12–23). CBD interacts directly with the serotonin_1A_ (5-HT_1A_) receptor (1, 24–27) and indirectly with the cannabinoid type 1 (CB_1_) receptor from the endocannabinoid (eCB) system by inhibiting the deactivation of endogenous cannabinoids (28–30). 5-HT_1A_ receptors and the eCB system regulate stress responses and can exhibit an anxiolytic effect when activated (27, 31–33). The CB_1_ receptor and its significance as a therapeutic target are currently under investigation (34, 35).

The pharmacological activity of the acidic forms of CBD and THC, cannabidiolic acid (CBDA) and Δ^9^-tetrahydrocannabinolic acid (THCA), has been scarcely reported so far (9). CBDA and THCA have been shown to interact with the eCB system with their functionality still under study (36–38). In addition to phytocannabinoids, cannabis plants contain terpenoid and flavonoid contents which are described to exhibit multiple effects, including anti-inflammation or sedation (39).

In the European Union (EU), companies declare their cannabis products for horses as “nutritional supplements” as opposed to medicinal products and are therefore not under regulation by the European Medicines Agency (EMA). To date, there is no authorized cannabis veterinary medicinal product in the EU or North America available (40). The Fédération Equestre Internationale (FEI) has banned all cannabis products due to the exhibition of potentially psychotropic effects (41). Since 2022, CBD is classified as a controlled medication (41).

In horses, options for the assessment of stress-responses include behavioral observations such as sedation scores or facial expression scales (42–46) as well as the analysis of physiological parameters like cortisol levels (47–51), heart rate and heart rate variability (48, 52–54). A common and frequently documented test to evaluate stress or fear in animals is the novel object test (6, 54–57). One report has assessed the effect of CBD in horses using a novel object test with evaluation of reactivity and heart rate after daily feeding of CBD pellets (dose: ~0.2 mg CBD/kg SID) for 6 weeks (6). When compared to a control group, reactivity scores were lower, but no significant difference in heart rate was identified (6).

Transportation and loading on trailers cause stress responses in horses which are reflected in increased heart rates and cortisol levels (58–60). Different training methods or even sedatives can be applied to effectively reduce these stress responses (58–61). No report has documented a potential effect of CBD on equine stress levels during loading on a trailer so far.

The aim of this study was to validate equine behavior and stress reactions including the response to a novel object test and a trailer test via heart rate and cortisol level monitoring in healthy horses following repeated oral administration of CBD containing paste (3 mg CBD/kg BID) for 15 days. The authors hypothesized that regular CBD administrations would have a calming effect in horses.

## 2 Materials and methods

### 2.1 Animals and study products

Twelve horses (seven mares and five stallions, Haflinger x Warmblood cross) were enrolled in the study. Horses were randomly assigned to a treatment or a control group (*n* = 6 + 6). Horses’ age was 3–16 years (median: 11 years) with an average body weight of 488 ± 55 kg in the treatment group. In the control group, the age was 10–26 years (median: 10.5 years) and the body weight 443 ± 56 kg. This study was designed as a prospective, randomized clinical trial. Study products were two pastes for oral administration, one containing 55% full spectrum CBD plant extract, medium-chain triglyceride (MCT) coconut oil, naturally occurring phytocannabinoids, terpenes, flavonoids and beeswax with a THC content of <0.2% (TAMACAN XL 55%^®^, Herosan healthcare GmbH, Austria). The second paste lacked an active ingredient and contained MCT coconut oil and beeswax [see part 1/2 for further detail (62)]. Pastes were labeled as “A” or “B” to conceal the formulation. The study was approved by the competent authority for licensing and notification procedures for animal experiments (LAVG) in Brandenburg, Germany (AZ: 2347-12-2021). Animals included had to pass a general physical examination by a licensed veterinarian and had a blood sample analysis including assessment of a complete blood count (CBC), kidney and liver biomarkers prior to study start. Exclusion criteria included irregularities during examination of the circulatory, respiratory and gastrointestinal systems, and signs of pain or inflammation such as fever and high white blood cell counts.

### 2.2 Multiple dose study

**Figure 1:**
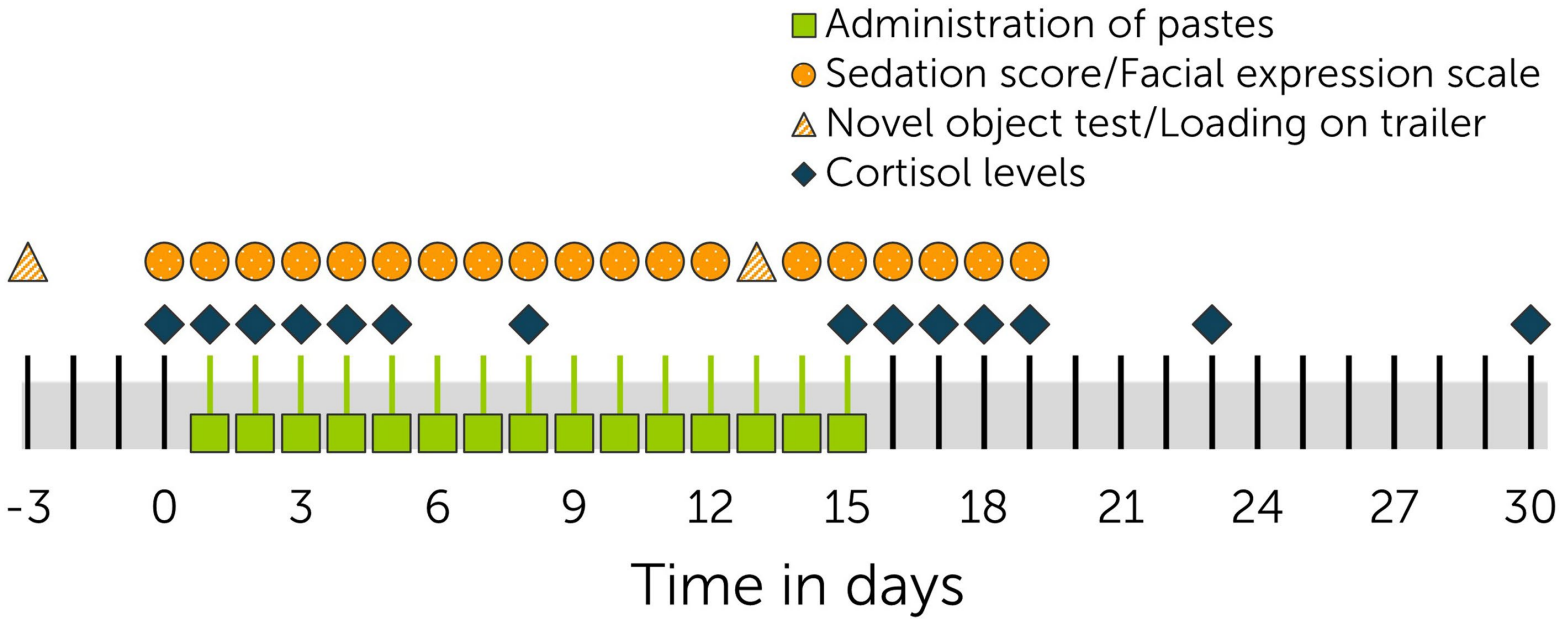
Timeline of multiple dose study. Pastes (3 mg CBD/kg and control) were administered twice daily (*n* = 6 + 6 horses) from days 1 to 15.

The multiple dose study started following a wash-out period of 25 days after the dose escalation study (62) to ensure a complete elimination of all cannabinoids following previous CBD applications. The day before study start, horses were physically examined, and a jugular vein catheter was aseptically placed. The jugular vein thrombophlebitis of one mare from the previous study part had resolved by this time (62). Serum and urine samples were tested for residual cannabinoid contents from the previous study part. Throughout the study, physical examination was repeated daily in every horse. Pastes (dose: 3 mg CBD/kg) were administered before feeding every 12 h (6:30 a.m. and 6:30 p.m.) for 15 days. Equine behavioral observations were video recorded daily between 7:30 am and 8:30 am using two acoustic stimuli (clicker and crackling of a plastic bag) and one visual stimulus (waving of a pink cloth). Video length was between 30 s and 60 s. Photographs of the horses’ faces were further taken once daily between 8:30 and 9:30 a.m. for assessment of facial expressions. Analysis of facial expressions was performed on one photo per horse and day. Videos and photographs were taken with an Apple iPhone SE^®^ (Apple Inc., CA, United States). Analysis of facial expressions was based on the facial sedation scale for horses (FaceSed) (43) and the Horse Grimace Scale (45). Facial parameters analyzed included orbital opening, position of ears, tension of chewing muscles represented by their visible presence, relaxation of lips and dilation of nostrils (62). Figure 1 shows a timeline of the study.

Blood and saliva samples obtained for assessment of cannabinoid levels (63) were additionally analyzed for cortisol levels. Samples were taken on the day before start of paste administrations (day 0), days 1–4, 8, 15–19, 23, and 30 (Figure 1). To avoid any influence of the circadian rhythm, only samples taken between 8:00 a.m. and 9:00 a.m. were chosen for cortisol analysis. Per each horse, 10 mL of blood was collected into serum separating tubes, stored at room temperature for 30–60 min and centrifuged at 3,000 × g for 10 min. From each tube, 5 mL of serum was then transferred into a fresh tube to be frozen and stored at −20°C. Samples were analyzed per each individual horse. To further analyze cortisol levels, saliva samples were taken with synthetic swabs (Salivette^®^, SARSTED AG & Co. KG, Nümbrecht, Germany). Swabs were removed from the tube using Gross-Maier Dressing Forceps and inserted into the horse’s mouth for approximately 30 s. Two to three swabs were used for each sample. Salivettes^®^ were centrifuged at 1,000 × g for 10 min. Saliva was subsequently transferred into new tubes, frozen and stored at −20°C.

### 2.3 Novel object test and trailer test

**Figure 2:**
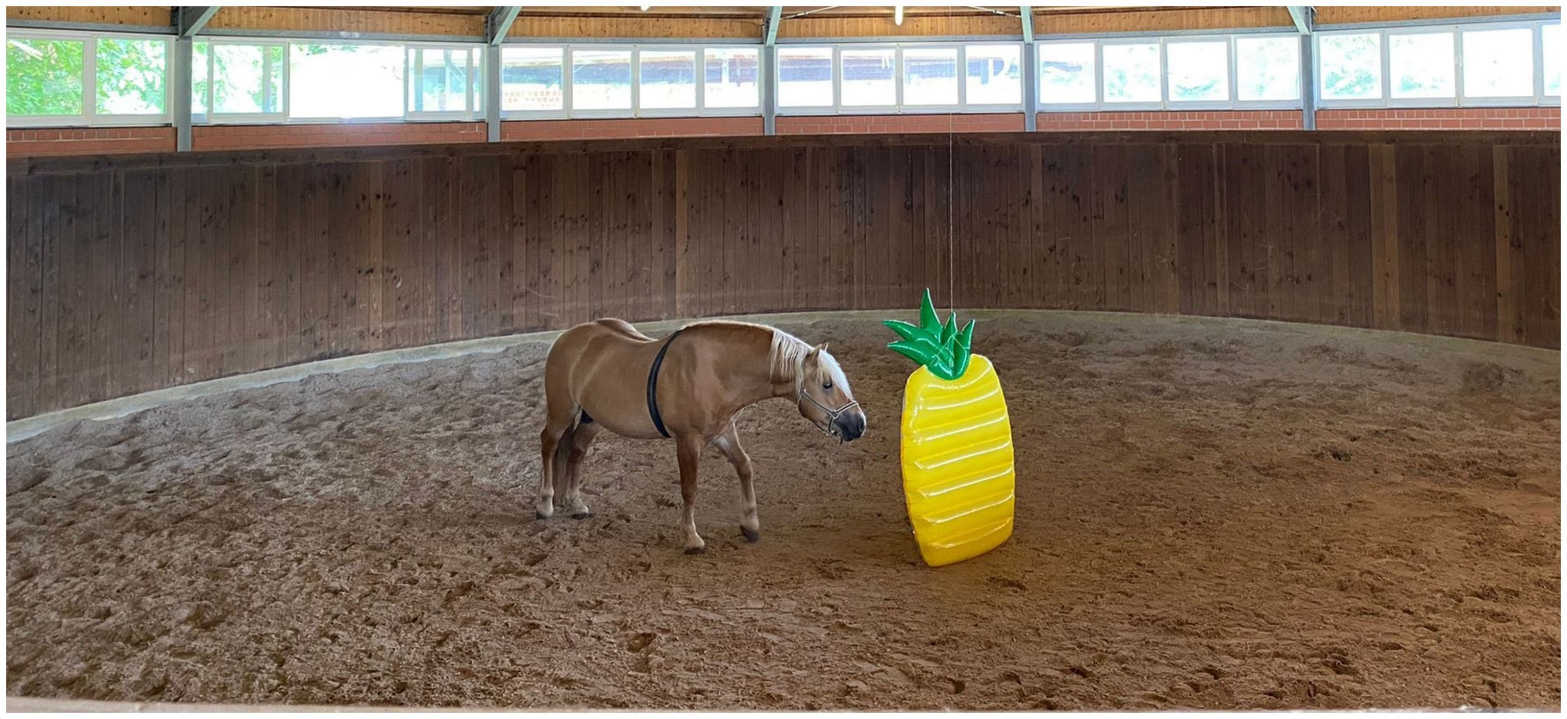
Novel object test. A pool raft (yellow pineapple) was chosen as the unknown object. The horse is wearing an electrode belt with a heart rate sensor around its chest.

To obtain baseline behavioral values, a novel object test and horses’ reactions to loading on a trailer were video recorded 3 days before the start of paste administration. Blood and saliva samples were taken for measurement of cortisol levels immediately prior to the novel object test. A Polar^®^ H10 heart rate sensor (Polar^®^ Electro Oy, Kempele, Finland) was attached to an electrode belt which spanned around the horse’s chest. Each horse’s coat was trimmed and moisturized with water over the heart base between the 4^th^ and 5^th^ intercostal space to enhance signal transmission. The heart rate sensor was connected to a mobile device via Bluetooth to record cardiac beat-to-beat (R-R) intervals using the Polar^®^ Equine App (Version 1.2.1, Polar^®^ Electro, Kempele, Finland). For the novel object test, an inflatable pool raft (approximately 170 × 80 × 10 cm, yellow pineapple) served as the unknown object. The pool raft was chosen for its bright and large exterior, and to minimize the possible risk of injury for the animals. The test began with horses being led into a round pen (Ø 15 m). The person leading the horse left the round pen and the object was lowered from the ceiling in the center of the round pen (Figure 2). After 10 min, the horse was taken out of the round pen and the object was raised to the ceiling again.

Each horse was subsequently led into a riding hall, where a trailer was parked. Horses were guided directly toward the trailer and up the ramp. If a horse was not willing to walk up the ramp, it was led back in a circle for another attempt (maximum five attempts). A second person was then asked to stand behind the horse and support its guidance toward the trailer. Loading was not enforced by any additional measures. After the tests, blood and saliva samples were obtained for later assessment of cortisol levels.

Both tests were repeated after 13 days of paste administration (Figure 1), as CBD concentrations in serum were expected to have reached a steady state by this time (63). A new pool raft with similar dimensions but differing outer appearance (green turtle) was chosen for the second novel object test. The remainder of the protocol including the setup for loading on a trailer remained the same. All tests were recorded using a video camera (GoPro HERO10^®^, San Mateo, United States).

#### 2.3.1 Assessment of novel object test

All video recordings were randomized and blinded. Evaluation was performed by one observer who was experienced in equine behavior studies and not aware of the horses’ group assignments. For each recording, the time periods spent in different movement patterns were assessed. Movement patterns included sniffing the ground, standing still, moving in each gait (walk, trot, canter) and rolling. During locomotion in each gait, the number of changes in direction were additionally documented. The horses’ reactions to the novel object itself were recorded by taking a note of the time it took a horse to first fixate the object visually, first approach the object and first touch the object.

#### 2.3.2 Assessment of trailer test

Randomized and blinded video recordings were assessed by an observer experienced in equine behavior studies, who was not involved in the previous study parts. Each horse’s compliance with entering the trailer was scored on a scale from 0 to 7 for each attempt (Table 1). The attempt with the highest score was selected for statistical analysis.

**Table 1:** Behavioral scoring for trailer test.

Score	
0	Horse stops in front of the ramp
1	One front leg is on the ramp
2	Both front legs are on the ramp (with support)
3	Both front legs are on the ramp (no support)
4	Both front legs are in the trailer (with support)
5	Both front legs are in the trailer (no support)
6	Horse is in the trailer (with support)
7	Horse is in the trailer (no support)

“Support” refers to a second person standing behind the horse to guide it on the trailer.

#### 2.3.3 Ethogram

An adjusted ethogram was developed to evaluate the behavioral traits shown throughout the novel object- and the trailer tests (Table 2). Randomized and blinded video analysis was performed by three observers who were not involved in the previous study parts but specifically trained for equine behavioral assessment. The number of behavioral traits displayed per horse was evaluated. Results of all three assessments were pooled to median values for further analysis.

**Table 2:** Ethogram developed for evaluation of the ^†^novel object test and ^§^trailer test.

Behavioral trait	Description
Bucking^†^	Fast dynamic movement in which the horse lowers its head, rounds its back and jumps in the air, sometimes leaving the ground with all four legs while kicking with the hindquarters
Cocking hindleg^†^	Horse standing firmly on three legs while one hindleg touches the ground with only the tip of the hoof
Defecating^†^	The horse relieving itself from fecal matter
Digging/scratching^†§^	Standing firmly on three legs while purposefully scratching the ground with the tip of one front hoof
Ear movement^§^	(Independent) flickering of one or both ears
Flehmen response^†^	Stretching the neck and the head upwards while curling the nose and exposing the teeth
Freezing^§^	Freezing of the horse with tense posture and forward gaze
Head tossing^†§^	Abrupt, powerful, short movement of the head and neck sideways or upwards; usually combined with tilting of the head
Licking/chewing^†^	Movement of the jaw that results in opening and closing of the mouth including movement of the tongue
Looking around or behind^§^	Turning the head and neck toward the back without leg movements
Neighing^†§^	The sound of a characteristic noise of a horse with different volumes and voice pitches
Remaining near exit^†^	The horse seeks close proximity to the exit of the round pen and remains there
Rolling^†^	Laying on the ground and demonstration a rolling motion, sometimes tilting over to the other side
Sniffing^†^	Horse lowers the head and sniffs the ground
Sniffing the ramp^§^	Horse lowers the head and sniffs the ramp
Snorting^†§^	Accelerated exhale through the nostrils accompanied by a characteristic flapping sound of the nostrils
Stomping^†^	Lifting of one leg and placing it back down forcefully
Tail swishing^†§^	Short, intense, omnidirectional movement of the tail
Treading on the spot^§^	Lifting and lowering the hooves without forward, backward or sideways movements
Urinating^†^	The horse relieving itself from urine in a characteristic stand
Walking backwards^§^	Stepping backwards
Walking sideways^§^	Stepping sideways

#### 2.3.4 Assessment of heart rate and heart rate variability

Each cardiac beat-to-beat (R-R) recording was divided into sections of 5 min as previously described (54). Automatic beat correction was applied to remove artifacts (threshold: very low, 0.3 s). Heart rate (HR) and heart rate variability (HRV) including the following parameters: mean HR in beats per minute (bpm), root mean square of successive beat-to-beat differences (RMSSD in milliseconds, ms) and standard deviation of normal-to-normal R-R intervals (SDNN, ms) were evaluated using the software Kubios^®^ HRV Standard (ver. 3.5, Kubios^®^ Oy, Kuopio, Finland).

### 2.4 Assessment of cortisol levels

Cortisol levels in serum and saliva samples were determined by means of high-performance liquid chromatography/tandem mass spectrometry (LC/MS/MS). Information on the sample preparation/extraction, instrumental conditions, validation, analysis and method validation are summarized in the Supplementary material.

### 2.5 Statistical analysis

Data were recorded in Microsoft Excel^®^ (Version 2304) and statistical analysis was performed with SPSS^®^ Statistics 27 (IBM^®^, NY, United States). Data were visually inspected and tested with a Shapiro–Wilk test for normal distribution. Behavioral observations (sedation score, facial expression scale) and cortisol concentrations were analyzed using an analysis of variance (ANOVA) with a Greenhouse–Geisser correction and a general linear model for repeated measures to test for differences between the treatment and the control group over time. Cortisol levels in serum and saliva were further tested for correlation using Spearman’s rank correlation coefficient.

For the novel object test and the trailer test, the differences between movement patterns, reactions to the unknown objects, scores for loading on a trailer, ethogram behavioral traits and cortisol levels during the first test (baseline) and after 13 days of paste administration were calculated for each horse. Differences between the treatment and control group were compared using a *t*-test (for normally distributed data) or a Mann–Whitney-U-Test (for not normally distributed data). For the ethogram, intraclass correlation coefficients determined the level of agreement between the observers for each observed behavioral trait. HR, RMSSD and SDNN parameters obtained during the second test were analyzed using an ANOVA to test for differences between the treatment and the control group. Residuals were visually inspected for normal distribution. The level of significance was *p* < 0.05.

## 3 Results

### 3.1 Animals

Daily physical examinations of all horses did not identify any side effects such as gastrointestinal intolerances associated with paste application. On the day before study start, no residual cannabinoid contents were detected in serum or urine. Regular blood analyses did not identify significant irregularities in CBC, kidney and liver biomarkers (63). CBD concentrations in serum reached a steady state after 2 days of CBD paste administration with a mean maximum serum concentration (C_max_) of 38.4 ± 8.9 ng/mL (63).

### 3.2 Behavioral observations

**Figure 3:**
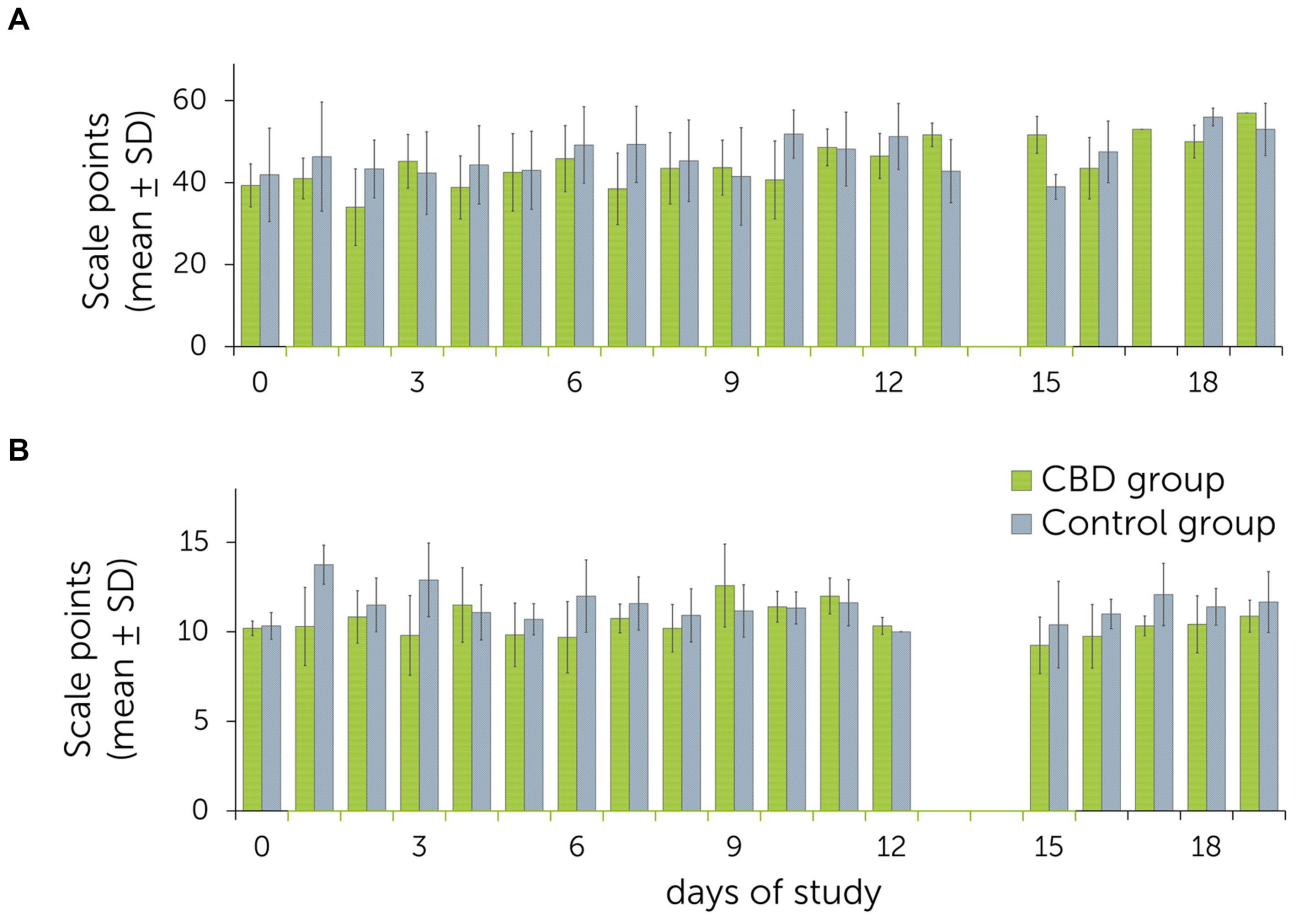
Mean ± standard deviations (SD) of behavioral observations obtained during the multiple dose study with daily administration of cannabidiol (CBD) and placebo pastes to a treatment and control group (*n* = 6 + 6 horses). The treatment group received CBD containing paste from days 1 to 15 (3 mg CBD/kg BID p.o.). **(A)** Summed up sedation scores after acoustic and visual stimulations (clicker, plastic bag, pink cloth). **(B)** Daily facial expression scores. Higher scale points relate to a higher level of relaxation/sedation.

Mean values for sedation scores ranged from 34.0 ± 5.0 (day 3) to 51.7 ± 1.5 (day 19) in the treatment group, and 39.0 ± 1.5 (day 15) to 56.0 ± 2.0 (day 19) in the control group. For the facial expression scale, values ranged from 9.7 ± 2.0 (day 3) to 12.6 ± 2.3 (day 9) in the treatment group, and 10.3 ± 0.8 (day 0) to 13.8 ± 1.1 (day 1) in the control group (Figure 3). On 12 out of 18 days, values for sedation scores were higher in the control group than in the treatment group. Comparison using an ANOVA with a Greenhouse–Geisser correction showed no significant differences between groups for the sedation score [*F*(3.0, 11.9) = 2.3, *p* = 0.127] and the facial expression scale [*F*(1.0, 1.0) = 1.5, *p* = 0.435]. Due to technical difficulties, videos and photographs of day 13 and 14 were not assessable for scoring.

### 3.3 Morning cortisol levels

**Figure 4:**
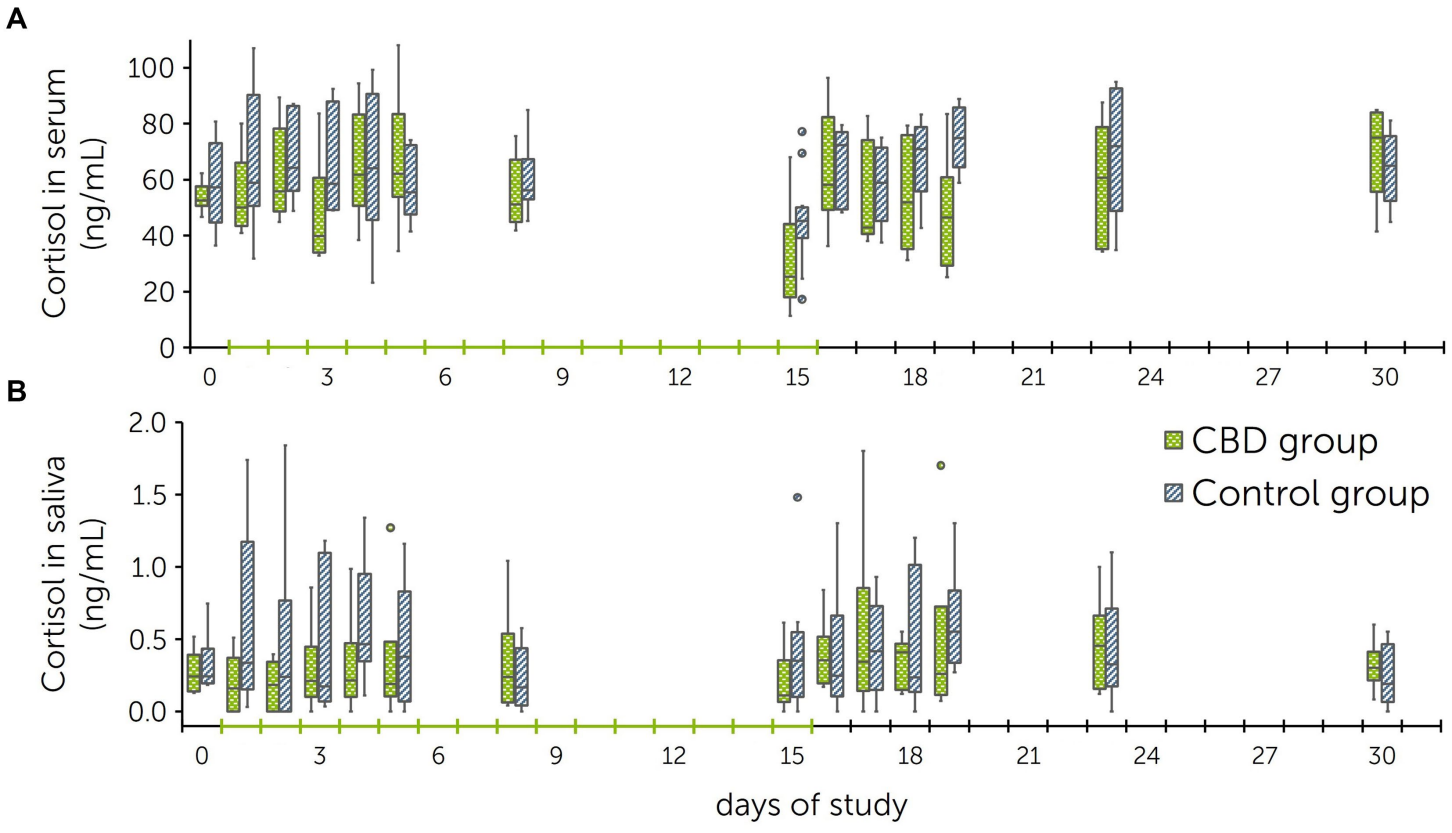
Boxplots of cortisol levels in serum **(A)** and saliva **(B)** obtained during the multiple dose study with daily administration of cannabidiol (CBD) and placebo pastes to a treatment and control group (*n* = 6 + 6 horses). The treatment group received CBD containing paste from days 1 to 15 (3 mg CBD/kg BID p.o.).

Throughout the course of the multiple dose study, cortisol levels in serum were on average 54.7 ± 18.6 ng/mL in the treatment group and 62.2 ± 19.2 ng/mL in the control group. For saliva, mean cortisol levels were on average 0.40 ± 0.30 ng/mL in the treatment group and 0.63 ± 0.45 ng/mL in the control group (Figure 4). Differences between groups were tested using an ANOVA with a Greenhouse–Geisser correction and were non-significant for cortisol levels in serum [*F*(4.1, 37.0) = 1.7, *p* = 0.171] and in saliva [*F*(1.6, 3.2) = 1.0, *p* = 0.442] over all days. Correlation between serum and saliva cortisol levels was *r*_s_ = 0.53 (*p* < 0.001).

### 3.4 Novel object test and trailer test

#### 3.4.1 Novel object test

**Figure 5:**
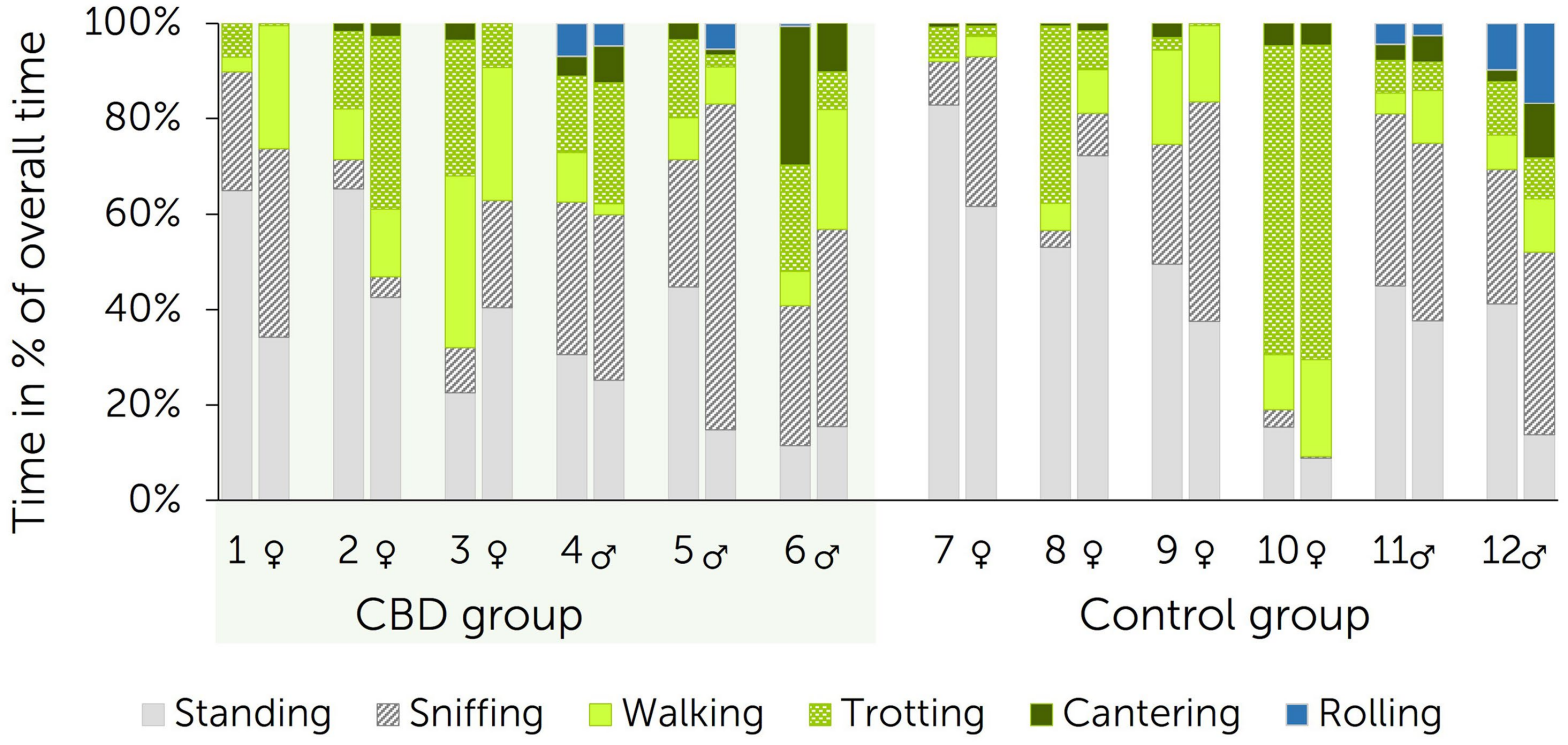
Movement patterns during novel object test in direct comparison per individual horse (1–12) between baseline (left bars) and after 13 days of paste administration (right bars) to a treatment and control group (*n* = 6 + 6 horses). The treatment group received a cannabidiol (CBD) containing paste twice daily from days 1 to 15 (3 mg CBD/kg).

The initial reactions to lowering of the pool raft was trotting or galloping alongside the outer parameter of the round pen in all horses. Movements then reduced to walking, standing or sniffing the ground with a subsequent continuation of trotting or galloping in a number of cases. Movement patterns for each individual horse are depicted in Figure 5. The difference between each movement pattern shown during the novel object test before trial start (baseline) and after 13 days of paste administration was calculated for each horse. Comparison of the differences between treatment and control group proved to be non-significant for all movement patterns (sniffing: *p* = 0.699; walking: *p* = 0.818; trotting: *p* = 0.818; galloping: *p* = 0.394; rolling: *p* = 0.699).

During both tests, horses changed direction several times. Differences in the number of changes of direction between before and after treatment ranged from 0 to 4 for each horse in the treatment group and from 1 to 8 for each horse in the control group. There was no significant difference found when compared between groups (*p* = 0.485).

In both novel object tests, all horses first fixated the pool raft visually 1.1–1.4 min after the start with non-significant difference between groups (*p* = 0.485). During the first novel object test (baseline), all horses approached the novel object after approximately 3 min (treatment group: 3.0 ± 1.3 min, control group: 3.0 ± 1.5 min). During the second novel object test, horses in the treatment group first approached the novel object after 4.4 ± 3.4 min and horses in the control group after 1.5 ± 0.5 min. Differences were non-significant (*p* = 0.065). During the baseline novel object test, four horses in each group touched the object. Two horses in the treatment group and four horses in the control group touched the pool raft during the second novel object test. Modes of touching included careful reaching with head and neck, tentative touching, or nibbling. Statistically significant difference was not identified between groups (*p* = 0.485).

##### 3.4.1.1 Novel object test: ethogram

Ten out of fifteen behavioral traits were rated with ICC values of > 0.90. The ICC value for “remaining near exit” was 0.80. “Cocking hindleg” and “stomping” were rated with ICC values between 0.50–0.75, and “licking/chewing” and “snorting” were rated with ICC values < 0.50.

In both groups, the most frequently exhibited trait was “sniffing” (treatment group: median at baseline = 12 times, median after paste administration = 16.5 times; control group: median at baseline = 9.5 times, median after paste administration = 10.5 times). Other behavioral traits (Table 2) were exhibited a median of 0–4 times. Individual stallions showed behavioral traits such as “tail swishing” and “head tossing” up to 18 and 29 times, respectively.

The difference between each behavioral trait exhibited during the baseline test and after paste administration was calculated per horse. Comparison of the differences between groups showed no significant effect [*p* values ranging from 0.132 (“head tossing”) to > 0.999 (“bucking”)].

#### 3.4.2 Trailer test

During the baseline test, three horses in the treatment group entered the trailer completely (scores 6 and 7, Table 1), one horse placed both front legs in the trailer (score 4), one horse went as far as putting both front legs on the ramp of the trailer (score 2) and one horse stopped in front of the ramp (score 0). In the control group, two horses entered the trailer (scores 6 and 7), two horses put both front legs in the trailer (scores 4 and 5) and two horses stopped before the ramp (score 0).

After 13 days of paste administration, the scores of six horses (three in each group) did not change (treatment group: scores 7, 7, 0; control group: scores 6, 0, 0). One horse in the treatment group was rated with a higher score (score 2 to 3). Two horses in the treatment group and three horses in the control group scored lower in the second test (treatment group: score 6 to 3, score 4 to 3; control group: score 7 to 6, score 5 to 3, score 4 to 3).

For each horse, the differences between scores determined during baseline and after paste administration were calculated with no significant effect when compared between groups (*p* = 0.589).

##### 3.4.2.1 Trailer test: ethogram

Observer agreement using the ICC was rated > 0.90 for six out of twelve behavioral traits. ICC values for “tail swishing,” “looking around or behind,” and “treading on the spot” were between 0.75 and 0.90. “Ear movement,” “freezing” and “snorting” were rated with ICC values of < 0.50.

In both groups, the behavioral trait most frequently observed was “ear movement” during the baseline test (treatment group: median of 5 times; control group: median of 3 times) and after paste administration (both groups: median of 3 times). “Ear movement,” “head tossing” and “looking around or behind” was mainly observed in stallions (between 10 and 13 times each). No horse exhibited “digging/scratching.” Differences were calculated between the baseline test and after paste administration for each individual horse. Differences were compared between groups using the Mann–Whitney-U-Test with resulting *p* values ranging from 0.180 (“looking around or behind”) to > 0.999 (“digging/scratching,” “neighing,” “walking sideways”).

#### 3.4.3 Heart rate and heart rate variability

Due to technical difficulties, recordings of R-R intervals during the novel object test and the trailer test before study start (baseline) were not available for analysis. It was decided to compare HR and HRV data obtained during the second tests between treatment and control group. The mean values assessed during the novel object test for HR were: 48.6 ± 1.5 bpm, for RMSSD: 93.4 ± 22.1 ms and for SDNN: 87.9 ± 26.3 ms in the treatment group. In the control group, mean values for HR were: 44.9 ± 5.3 bpm, for RMSSD: 113.8 ± 36.5 ms and for SDNN: 113.5 ± 58.9 ms.

During the trailer test, the mean HR was 47.2 ± 3.7 bpm, mean RMSSD was 121.1 ± 21.3 ms and mean SDNN was 118.6 ± 37.6 ms in the treatment group. In the control group, mean values were HR: 46.3 ± 10.7 bpm, RMSSD: 124.2 ± 45.0 ms and SDNN: 132.4 ± 61.0 ms. Analysis using a one-way ANOVA with a Greenhouse–Geisser correction found no statistically significant differences between treatment and control group over both trials for HR: *F*(1.5, 12.2) = 1.2, *p* = 0.312, RMSSD: *F* (5, 40) = 1.6, *p* = 0.183 and SDNN: *F* (6, 36) = 1.6, *p* = 0.178.

#### 3.4.4 Cortisol levels

**Figure 6:**
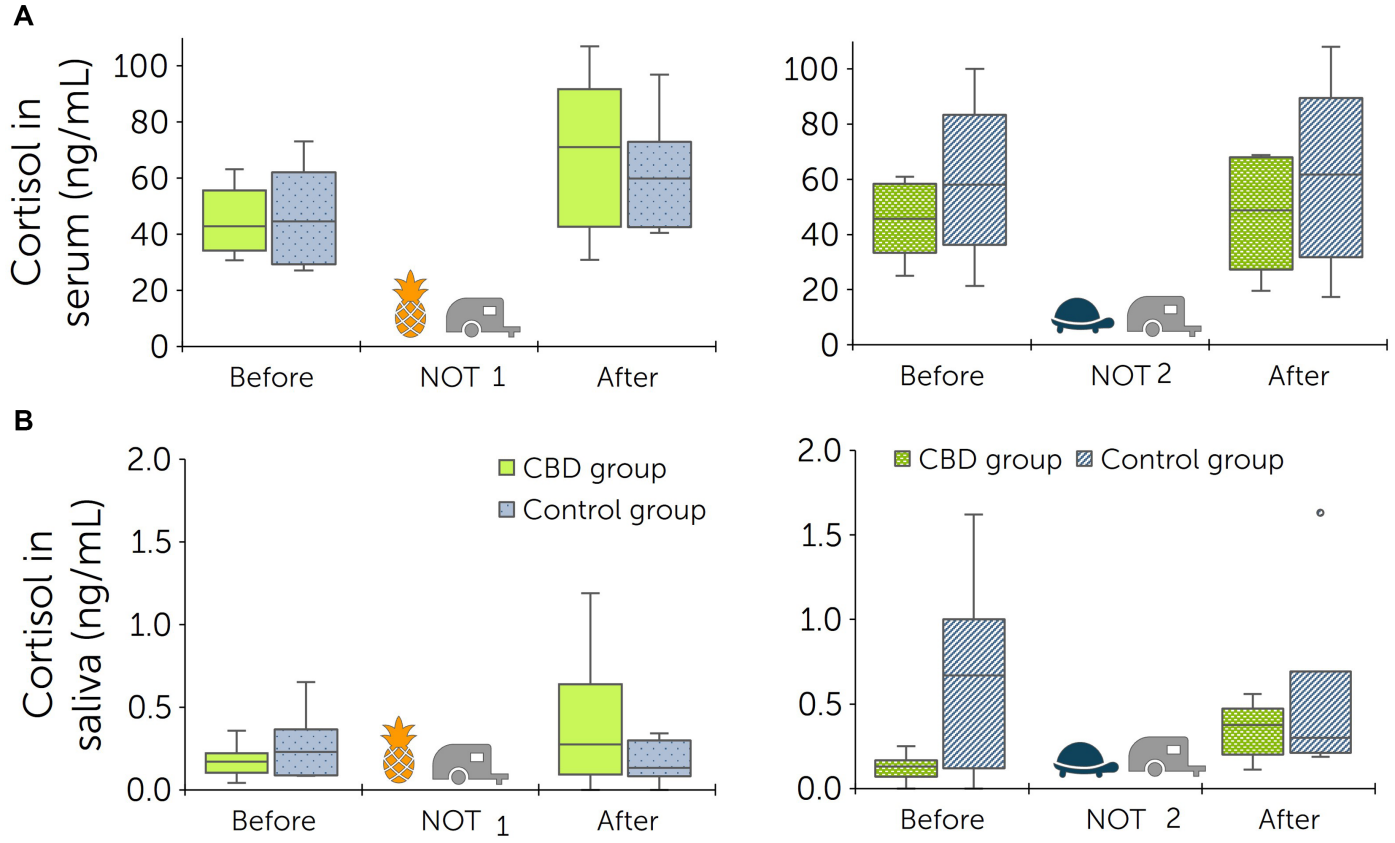
Cortisol levels in serum **(A)** and saliva **(B)** before the novel object test (NOT) and trailer test, and immediately after both tests. Tests were performed twice: prior to start of paste administrations (baseline) and following 13 days of paste administrations to a treatment and control group (*n* = 6 + 6 horses). Pool rafts were used as novel objects [pineapple for the baseline test (NOT 1), turtle for the second test (NOT 2)]. The treatment group received a cannabidiol (CBD) containing paste twice daily from days 1 to 15 (3 mg CBD/kg).

Serum and saliva samples for cortisol analysis were obtained prior to each novel object test and after each trailer test. Before the first novel object test (baseline), cortisol levels of horses in the treatment group were 44.68 ± 11.08 ng/mL in serum and 0.17 ± 0.09 ng/mL in saliva. After the baseline tests, cortisol levels increased to 68.87 ± 24.95 ng/mL in serum and 0.46 ± 0.38 ng/mL in saliva. Before the second novel object test, serum cortisol levels were 45.22 ± 12.61 ng/mL and saliva cortisol levels 0.15 ± 0.05 ng/mL. After the second trailer test, cortisol levels increased to 47.23 ± 18.27 ng/mL (serum) and 0.35 ± 0.15 ng/mL (saliva) (Figure 6).

Prior to the baseline novel object test, cortisol levels in the control group were 46.28 ± 16.10 ng/mL in serum and 0.26 ± 0.19 ng/mL in saliva. After loading on a trailer, cortisol levels reached 60.87 ± 18.67 ng/mL in serum and 0.20 ± 0.09 ng/mL in saliva. Before the second novel object test, serum cortisol levels were 59.40 ± 25.12 ng/mL and saliva cortisol levels were 0.78 ± 0.48 ng/mL. After the second trailer test, cortisol levels were 61.42 ± 30.30 ng/mL (serum) and 0.50 ± 0.51 ng/mL (saliva) (Figure 6).

Differences between cortisol levels measured in serum and saliva before and after the tests were calculated for each horse. Comparison of test results from the second tests found a significant difference between groups for cortisol levels in saliva (*p* = 0.016), but not in serum (*p* > 0.999). Within the treatment group, comparison between baseline tests and tests following CBD paste administration showed no significant differences (serum: *p* = 0.505; saliva: *p* > 0.999).

## 4 Discussion

Regular oral administration of a CBD containing paste at a dose of 3 mg/kg was well-tolerated by all horses in this study. Multiple oral CBD administrations did not have a significant effect on behavioral observations and cortisol monitoring. Parameters investigated in a novel object test and during loading on a trailer did not differ significantly from the control group.

Case reports have described CBD as an effective agent for the treatment of mechanical allodynia, chronic crib-biting and wind-sucking at an oral dose of 0.5 mg CBD/kg BID in horses (64, 65). These reports did not test CBD levels in serum, but previous studies reported maximum CBD concentrations of less than 20 ng/mL in serum following administration of up to 3 mg CBD/kg p.o. (8, 66–71). Two studies found C_max_ levels of 51 ng/mL CBD in serum following oral administration of 2 mg CBD/kg SID for 7 days (67, 70), and C_max_ levels of 55.7 ng/mL CBD in serum following a single oral dose of 10 mg CBD/kg (72). The C_max_ levels of 38.4 ± 8.9 ng/mL in serum reported during the current study (63) are therefore in line with previous reports, and comparatively high (70). In dogs, similar CBD dose levels lead to much higher concentration maxima in serum: one study has shown that the median C_max_ of CBD was 102.3 ng/mL after single oral administration of 2 mg CBD/kg (4). The absorption and retention of CBD in horses seems to be more akin to humans than dogs (70). Single oral intake of 400 mg CBD resulted in a subjective reduction in anxiety in humans with generalized social anxiety disorder (15). However, as no therapeutic serum concentrations for anxiety in humans are available so far, further studies are required to translate administered CBD dose levels to therapeutic serum concentrations.

The facial expression scale used in this study was based on the facial sedation scale for horses (FaceSed) and the Horse Grimace Scale (HGS) (43, 45). Two studies have reported an effective assessment of facial expressions using the HGS to indicate pain levels (73, 74). In the current study, daily behavioral observations of sedation levels using a sedation score and a facial expression scale did not differ significantly between treatment and control group. This assessment is in line with previous studies that found no significant effect on sedation levels following regular CBD pellet feedings (~0.29 mg CBD/kg over 56 days) in horses (7) and oral administration of CBD treats (4.5 mg CBD/kg BID over 21 days) in dogs (18). Reports on US veterinarians and pet owners’ perceptions of CBD and hemp use in dogs state that sedation/tiredness were the most commonly observed side effects (75–77). In humans, sedation was reported as a side effect following daily oral intake of 600 mg CBD over 6 weeks (78). As doses were higher in these reports, the question remains whether increased dose levels and therefore increased serum concentrations would lead to a similar effect in horses.

Cortisol is a steroid hormone which is subject to a circadian rhythm. Cortisol levels assessed in previous publications were reported to be highest between 8 am and 12 pm (serum: 25–70 ng/mL; saliva: 0.55–0.70 ng/mL) (50, 79) and are comparable to levels reached in the current study. Depending on the time of day and stress exposure, saliva levels can reach up to 3 ng/mL in horses but usually stay below 1 ng/mL (49, 50, 80). Saliva sampling is a noninvasive, pain-free additional technique to gain more information about cortisol levels (49, 81). Salivary and serum cortisol levels have been reported to have different degrees of correlation (*r*_s_ = 0.32–0.80) (50, 81). In this study, a moderate correlation was seen between serum and salivary cortisol levels (*r*_s_ = 0.53) (82). Minor disruptions leading to stress responses can result in deviations from the normal circadian cortisol rhythm and may elevate cortisol levels in blood (50, 79). In this study, no significant effect of CBD on morning cortisol levels was identified.

Novel object tests have been used in a variety of species and can be performed with different unknown objects (54–57) or even unknown horses (Novel horse test) (83). Novel object tests are designed as fear tests and are used to document the intensity of an animal’s fearfulness when confronted with the unknown object. As no standard protocol exists, neither regarding the kind of object nor the duration of exposure, scoring of reactions and assessment of additional parameters (such as heart rate) tend to vary. In this study, two novel object tests were performed with similarly sized yet differently colored and shaped objects (pool rafts: yellow pineapple and green turtle) to make the test results comparable and exclude a habituation effect. One report tested habituation to a frightening stimulus (white nylon bag) in 2-year-old colts. It was concluded that the horses were habituated to the stimulus after four training sessions which were all conducted within 1 day (84). As the novel object tests performed in this study were only performed twice and were 16 days apart, habituation was considered to be an unlikely limiting factor. The effect of CBD in horses has been tested in another study using a novel object test following daily oral administration of CBD pellets (~0.2 mg CBD/kg) (6). A significantly lower degree of reactivity compared to a control group was documented (6). A fear response test performed in dogs following oral CBD treatment (1.4 mg CBD/kg) showed no significant effect (85). In agreement with this report, the current study found no significant difference between treatment and control group regarding movement patterns. Reaction times to the novel object differed between groups: during the first novel object test, horses in both groups took about 3 min to first approach the novel object. During the second test, horses in the treatment group took more time to first approach the object (4.4 ± 3.4 min) than horses in the control group (1.5 ± 0.5 min). These differences could suggest that CBD does either not exhibit a fear-reducing effect in the studied dose level, or that CBD has a relaxing effect and reduces the horse’s interest in the novel object. Statistical analysis showed that the differences between groups are bordering on significance (*p* = 0.065), which might be biased by the small sample size. Future tests should include larger sample sizes and potentially nervous horses when determining CBD’s effect as a fear-reducing or anxiolytic agent.

Loading on a trailer is considered a stressful event for horses (58–60). Different training methods are described to reduce horses’ discomfort and anxiety (58–60). In addition to training, sedatives like acepromazine may be used to reduce stress responses (61). Oral CBD (total of 400 mg, single administration) has been reported to subjectively decrease anxiety in humans with generalized social anxiety disorder (15). The effect of CBD on horses’ reactions to loading on a trailer has not been reported yet, but results of this study suggest that it does not increase horses’ willingness to enter a trailer at the tested dose level.

Behavioral traits displayed by horses during the novel object- and the trailer test were assessed using a customized ethogram. Behavioral observations may be performed using a software (53) or handwritten lists prepared by one to four independent observers (73, 74, 86). To reduce subjectivity, three observers rated behavioral traits in this study. Most behavioral traits displayed a good (0.75–0.90) to excellent agreement (> 0.90) (87). Behavioral traits with poor agreement (< 0.50) included “ear movement,” “freezing,” “licking/chewing” and “snorting.” Poor scores might be related to an insufficient description of the respective traits, or to the more difficult detection of smaller movements such as “ear movement” or “licking/chewing” especially in combination with other movements when watching a video recording. A wide variety of behavioral traits were assessed including noises (“neighing”) and whole body movements (“walking backwards”), as well as behaviors indicative of stress such as “bucking” or “head tossing” (88). No significant differences in displayed behavioral traits were identified between treatment and control group.

Studies investigating heart rate (HR) and heart rate variability (HRV: RMSSD and SDNN) have shown that a decrease in HR and increase in RMSSD and SDNN suggest an autonomic shift toward a parasympathetic dominance and are therefore indicative of the horse’s stress levels (48, 54, 89–92). Measurement of HR and HRV is an established tool to evaluate stress responses due to pain or anxiety-inducing events (90, 93–96). Additionally, assessments of HR and HRV have been performed during novel object tests (54–56, 97), and loading on a trailer and subsequent transport (98, 99) in horses. The effect of CBD on HR and HRV has been documented in horses, dogs, humans and rodents with varying results. In horses, HR assessed during a novel object test found no significant effect between a treatment group fed 100 mg pelleted CBD (~0.2 mg CBD/kg) and a control group (6). A stress test performed in dogs similarly found no significant differences in HR and HRV values between a treatment (single oral administration of 4 mg CBD/kg) and a placebo group (100). A second report in dogs equally identified no significant changes in RMSSD and SDNN following a fear response test when treated orally with 1.4 mg CBD/kg (85). In contrast, single intraperitoneal CBD administration in rodents (10 mg CBD/kg) significantly reduced the increase of HR and blood pressure in a stress inducing and fear conditioning setting, suggesting an anxiolytic effect (14, 16). In this study, HR values were higher and RMSSD and SDNN were lower in the treatment than in the control group, indicating a less pronounced parasympathetic state in the treatment group. However, as these differences were statistically non-significant, their relevance is debatable.

Measurement of cortisol concentrations is an established parameter for stress evaluation in horses (49, 51, 81, 92, 99). When comparing the cortisol levels before and after the novel object- and trailer tests, cortisol levels in serum increased to varying degrees (Figure 6). Within the treatment group, the increase was less pronounced after the second round of tests. Statistical analysis showed that this reduction was non-significant. In the control group, salivary cortisol levels had decreased after both test rounds. The difference between treatment and control group was therefore found to be significant (*p* = 0.016). The effect of CBD on cortisol levels has been investigated in humans, dogs and horses with varying results (17, 66, 100–102). After a stress test, dogs that received oral CBD (4 mg CBD/kg) showed significantly lower serum cortisol concentrations than a control group (100). In horses, one study compared cortisol levels between horses that were administered CBD oil and horses receiving olive oil after transportation with no significant findings (66). Studies performed in humans are difficult to compare due to their differing designs and intentions, but have similarly not found a significant effect of CBD on cortisol levels (101, 102).

As all cannabinoids are listed as prohibited substances by the FEI, and CBD is defined as a controlled medication (41), future studies are required to determine what effects oral dosing of CBD exactly exerts in horses, and what dose levels and intervals are needed to achieve these effects. No consistently significant effects on equine behavior were observed in this study.

A small sample size is the main limitation of this study. Further limitations include the missing recordings of R-R intervals during the novel object test and the trailer test before study start (baseline). Consequently, comparison of HR and HRV was carried out between groups following paste administration. Subjects were healthy horses that did not show behavioral problems. Further trials with larger sample sizes are needed to validate the potential effectiveness of CBD in anxious or nervous horses. Future studies may also include more detailed assessments of HRV parameters including the parasympathetic tone activity (PTA) index. Oral dosing using different formulations such as micellar formulation should also be considered (72). Clinical studies as have been performed with dogs (4) are of interest to further assess the potential use of CBD in equine medicine.

## 5 Conclusion

This study did not detect consistently significant effects of regularly administered oral CBD (3 mg/kg BID over 15 days) on behavioral observations or morning cortisol levels in healthy horses. Horses’ reactions to a novel object and loading on a trailer were tested with no significant differences identified between treatment and control group. Parameters assessed included movement patterns, reaction to the novel object, heart rate and heart rate variability, and cortisol levels in serum and saliva. No adverse reactions were observed following multiple administrations of a CBD containing paste. Further research is required to determine adequate indications for the use of CBD products in horses.

## Data availability statement

The raw data supporting the conclusions of this article will be made available by the authors, without undue reservation.

## Ethics statement

The animal study was approved by the competent authority for licensing and notification procedures for animal experiments (LAVG) in Brandenburg, Germany (AZ: 2347-12-2021). The study was conducted in accordance with the local legislation and institutional requirements.

## Author contributions

FE: Conceptualization, Data curation, Formal analysis, Investigation, Methodology, Project administration, Software, Validation, Visualization, Writing – original draft. AE: Conceptualization, Data curation, Formal Analysis, Funding acquisition, Investigation, Methodology, Project administration, Resources, Supervision, Validation, Writing – review & editing. MM: Formal analysis, Investigation, Methodology, Project administration, Software, Validation, Writing – review & editing. KCJ: Formal analysis, Methodology, Software, Validation, Writing – review & editing. SW: Formal analysis, Methodology, Software, Validation, Writing – review & editing. NB: Conceptualization, Data curation, Methodology, Project administration, Writing – review & editing. JB: Data curation, Formal analysis, Investigation, Methodology, Project administration, Writing – review & editing. MP: Data curation, Formal analysis, Investigation, Methodology, Writing – review & editing. MT: Methodology, Supervision, Writing – review & editing. WB: Conceptualization, Methodology, Project administration, Supervision, Writing – review & editing. CL: Conceptualization, Funding acquisition, Methodology, Project administration, Resources, Supervision, Writing – review & editing. MW: Conceptualization, Investigation, Methodology, Project administration, Resources, Supervision, Writing – review & editing.
