# Supplementary material for: Behavioral observations, heart rate and cortisol monitoring in horses following multiple oral administrations of a cannabidiol containing paste (part 2/2)
Source: Front Vet Sci. 2024 Jan 3;10:1305873. doi: 10.3389/fvets.2023.1305873 (PMC10791836; doi:10.3389/fvets.2023.1305873)
Supplement: Supplementary file 1 [file Data_Sheet_1.PDF]

SUPPLEMENTARY MATERIAL

1 Assessment of cortisol levels

1.1 Preparation of serum and saliva samples for cortisol analysis

Aliquots of 0.5 mL serum or saliva were fortified with 25 ng/mL or 0.5 ng/mL of the internal standard D4-hydrocortisone, respectively. After pH adjustment to 9.6 with a 2:1 mixture of solid $NaHCO_3/K_2CO_3$, samples were extracted with 5 mL *tert*-butyl methyl ether (tBME) for 20 minutes on a horizontal shaker. Centrifugation for 5 min at 600 g enabled the separation of the ethereal layer, which was evaporated. The residue was reconstituted in 2 mL $MeOH/H_2O$ (95/5, v/v). The methanolic layer was washed with 5 mL *n*-pentane for 5 minutes and the supernatant was separated by centrifugation and discarded. The methanolic layer was evaporated and the residue reconstituted in 100 µL LC buffer consisting of ammonium acetate (5 M)/acetonitrile (3/2, v/v) and 1% acetic acid. Aliquots of 10 µL were injected into the LC-MS/MS instrument.

1.2 High performance liquid chromatography/tandem mass spectrometry (LC/MS/MS) for detection of cortisol in serum and saliva samples

LC-MS/MS analyses were performed on an Agilent Series 1260 liquid chromatograph (Waldbronn, Germany) coupled to a 5500 QTrap triple-quadrupole mass spectrometer (AB Sciex, Darmstadt, Germany) equipped with an electrospray ionisation (ESI) interface. The column was a Nucleodur C18-Pyramid-column with dimensions of 2 x 50 mm and particle size of 3 µm protected by a guard column from Macherey-Nagel (Düren, Germany). The LC conditions were as follows: mobile phase A = ammonium acetate buffer (5 mM, pH 5, containing 0.1% acetic acid), B = acetonitrile, flow rate 0.35 mL/min, gradient 0% B → 100% B in 7 minutes, re-equilibration time 4.5 minutes at 0% B. Samples were measured in the negative operation mode at an interface temperature of 450 °C with an ion spray voltage (ISV) of -4500 V. Diagnostic ions of the analytes were generated by collision induced dissociation (CID) with nitrogen at a collision gas pressure of 2.3 x 10-3 Pa. Multiple reaction monitoring (MRM) experiments were performed on the most abundant ion transitions, which were optimized by support of the software Analyst 1.6 after infusion of the corresponding reference solutions. Selected quantifier MRM transitions were m/z 421/282 and 425/335 for the hydrocortisone acetate adduct and the D4-hydrocortisone acetate adduct, respectively.

1.3 Method validation for the analysis of cortisol

Validation for the quantification of cortisol in plasma samples was conducted considering precision and accuracy, stability, lower limit of detection (LLOD), lower limit of quantification (LLOQ), linearity, selectivity and robustness. A separate validation for cortisol in saliva was not performed. Instead, a calibration line was individually prepared for each batch of post administration samples and used for calculation of the cortisol concentrations within this batch. Cortisol was identified by three specific ion transitions. Additionally, the presence of cortisol was confirmed by the product ion scan of the molecular ion ($M - H^+$) of its acetate adduct. Ten different serum samples showed no interfering signals at the retention time of (endogenous) cortisol that could interfere with the signal identification and peak integration of cortisol.

Precision, accuracy and stability were determined as described for the validation of cannabinoids (47). Table 1 summarizes the results with respect to the cortisol concentration levels selected for the

tested validation parameters. A signal-to noise ratio of 3 and 9 was used to determine LLOD and LLOQ in equine serum and saliva, respectively. A series of 6 determinations at the concentration of the LLOQ was used for verification. The linearity of cortisol in serum was examined by a series of 9 different concentrations spiked into a cortisol stripped serum (SeraSub™, CST Technologies, Great Neck, USA). Linearity of cortisol in saliva was derived from 8 calibrators with water as the surrogate matrix for saliva. A weighting factor of 1/x was selected for both calibration lines. Correlation factors (R^2) were > 0.98 for both calibration curves and measured concentrations remained within the acceptance range of 85 - 115% of the theoretical cortisol concentration. Robustness was determined at a concentration of 20 ng/mL following the validation design for cannabinoids (47). All ten serum samples showed signals for cortisol with reproducible ion ratios. Relative retention time shifts were within acceptable ranges below 0.8%.

Table 1: Validation results for cortisol.

**Analyte**	**Matrix**	**LLOD [ng/mL]**	**LLOQ [ng/mL]**	**Intra-day Precision CV [%] at 1/20/100 ng/mL**	**Inter-day Precision CV [%] at 1/20/100 ng/mL**	**Accuracy RE [%] at 1/20/100 ng/mL**	**Stability [%] at 20 ng/mL**
Cortisol	Serum	0.1	0.2	3.8/6.7/5.7	4.7/5.4/11.0	3.3/4.0/-6.5	96
	Saliva	0.02	0.05	n.a.	n.a.	n.a.	n.a.

Abbreviations: n.a.: not applicable.
